# Systems pharmacology-based optimization of Ma Xing Shi Gan components for the enhanced treatment of chick health issues caused by infectious bronchitis virus

**DOI:** 10.3389/fcimb.2025.1585293

**Published:** 2025-08-07

**Authors:** Huixin Liu, Yang He, Chenchen Wang, Xiaofang Wei, Jiayi Chen, Kaijun Wang

**Affiliations:** ^1^ Hunan Provincial Key Laboratory of the Traditional Chinese Medicine Agricultural Biogenomics, Changsha Medical University, Changsha, China; ^2^ Animal Experiment Center of Sichuan Academy of Chinese Medicine Sciences, Chengdu, China; ^3^ College of Animal Science and Technology, Guangxi University Key Laboratory of Animal Breeding, Disease Control and Prevention, Nanning, China; ^4^ College of Veterinary Medicine, Hunan Agricultural University, Changsha, Hunan, China

**Keywords:** infectious bronchitis virus, traditional Chinese medicine inheritance computing platform, vitro and *in vivo* experiments, liquid chromatography-mass spectrometry, network pharmacology

## Abstract

**Introduction:**

Infectious bronchitis virus (IBV) imposes severe economic burdens on the poultry industry, and current treatments face challenges in efficacy and sustainability, necessitating the development of novel therapeutic strategies. To address this, this study employed the Traditional Chinese Medicine Inheritance Computing Platform (TCMICS) to collect clinical prescriptions for IBV treatment, based on which two optimized versions of the traditional Chinese medicine Maxing Shigan Decoction (MXSG), namely MXSG-mix1 and MXSG-mix2, were designed. In vitro cell culture and in vivo chicken model experiments were then carried out, including egg testing, clinical symptom observation, immune function analysis, and viral load quantification, to assess the antiviral activity of the optimized formulations.

**Methods:**

To explore the underlying mechanisms, liquid chromatography-mass spectrometry (LC-MS) was combined with network pharmacology to identify 28 active compounds in MXSG-mix and 47 key genes involved in viral replication, inflammation, and apoptosis pathways. Furthermore, molecular docking and RT-qPCR were performed, which confirmed that MXSG-mix downregulated BCL2 expression and interacted with AKT1 and CASP3, thus inhibiting IBV-induced cell apoptosis.

**Results and discussion:**

The results showed that both MXSG-mix1 and MXSG-mix2 exhibited superior anti-IBV activity compared to traditional MXSG, effectively reducing viral load and improving immune responses in vivo. In conclusion, integrating TCMICS, LC-MS, and network pharmacology offers a novel paradigm for developing veterinary TCM formulations. The optimized MXSG-mix shows potential as an effective, multi-target therapeutic against IBV, providing valuable insights for future anti-viral drug development in poultry medicine.

## Graphical Abstract

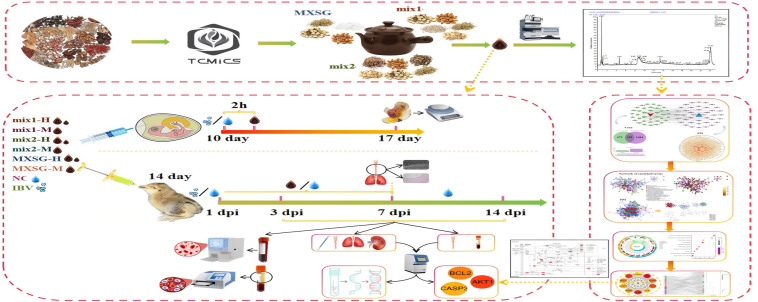

## 1 Introduction

Infectious bronchitis (IB) is an acute and highly contagious respiratory ailment caused by the infectious bronchitis virus (IBV) from the Gammacoronavirus genus, which belongs to the family Coronaviridae and order Nidovirales. This disease affects chickens of various breeds and ages, manifesting as tracheitis, conjunctivitis, and ciliary stasis (a loss of ciliary activity) in the respiratory tract. It can lead to decreased weight gain in poultry, lower egg production, reduced eggshell quality, impaired feed efficiency, as well as increased false laying hens and secondary bacterial infections. Consequently, it imposes a significant economic burden on the global poultry industry (Shirvani et al., 2020). However, due to the high variability and diversity of IBV strains, prevention and treatment of this disease pose considerable challenges often leading to immunization failure. In response to previous failures and concerns regarding drug residues, the Chinese government has implemented a prohibition on the use of antivirals in food animals within China. Traditional Chinese Medicine (TCM) offers unique benefits in enhancing physiological functions, boosting immunity, improving disease resistance, and increasing antiviral capacity in animals. Therefore, exploring and applying antiviral compounds derived from traditional Chinese medicine could potentially serve as an alternative or supplementary strategy for IBV vaccination. Numerous reports have documented the efficacy of traditional Chinese medicine in treating IBV, such as Hypericum aculatum (Chen et al., 2019b), garlic (Mohajer Shojai et al., 2016), Houttuynia cordata (Yin et al., 2011), and Gandilone compound granules (SGDL) (Feng et al., 2021). Furthermore, the studies investigating the utilization of Chinese traditional medicine for respiratory diseases, including COVID-19, have shown promising results.

The Maxingshigan Decoction (MXSG), a traditional Chinese compound comprising liquiritin, glycyrrhetinic acid, amygdolin, ephedrine, pseudoephedrine, and methylephedrine, has been used for centuries in China to treat respiratory diseases (Chen et al., 2020; Jiang et al., 2015; Tsai et al., 2017; Wei et al., 2020). MXSG has demonstrated efficacy against viral respiratory diseases and recently exhibited potential in the treatment of COVID-19 (Yang et al., 2020; Shao et al., 2020; Hsieh et al., 2012; Li et al., 2021; Zhang et al., 2021). The respiratory diseases caused by IBV share similarities with those caused by SARS-CoV-2, the virus responsible for COVID-19 (Nefedova et al., 2021). MXSG has been successfully utilized in treating IBV in Chinese chicken farms since the 19th century. New herbal formulas, such as Jinhua Qinggan granule (Zhu et al., 2023) and Maxing Shigan Weijing decoction (Zeng et al., 2020), which are derived from MXSG, have also demonstrated efficacy against coronaviruses. However, due to the adaptable nature of traditional Chinese medicine prescriptions, analyzing the fundamental principles of medication poses challenges. There is a lack of in-depth research on both macro and micro levels regarding the established scheme and mechanism of prescription Chinese medicine (Yu et al., 2022). The integration of the TCM Inheritance Computing platform (TCMICS V3.0) with network pharmacology can facilitate a deeper exploration into the medication principles underlying traditional Chinese medicine prescriptions for respiratory diseases (Dai et al., 2018). This approach can assist in summarizing prescription rules, generating novel prescriptions, and elucidating potential mechanisms of action for traditional Chinese medicine compounds. Such research will contribute to confirming the effectiveness and therapeutic advantages of these compounds over conventional prescriptions.

The objective of this study is to evaluate the *in vivo* anti-IBV activity and mechanism of action of a modified MXSG decoction, derived from modern Chinese medicine data mining techniques on Specific Pathogen Free (SPF) chicken embryos and chicks. This novel formulation originates from MXSG, which is renowned for its potential antiviral properties against IBV coronavirus. This study represents pioneering research in the field of Chinese veterinary medicine based on TCMICS V3.0 and network pharmacology. The preventive and therapeutic effects of the MXSG mixture were evaluated through clinical efficacy assessment, immune organ index measurement, humoral immunity analysis, cellular immunity examination, and viral RNA content determination. The mechanism underlying the anti-IBV effect of MXSG-mix was confirmed using network pharmacology and identification of key targets. The results demonstrated a significant inhibitory effect of the MXSG mixture against IBV infection. Furthermore, both additive and subtractive formulations of MXSG exhibited promising inhibitory effects on IBV infection, highlighting their potential as innovative anti-IBV drugs.

## 2 Method

### 2.1 Virus and chicken embryos

The pathogenic IBV M41 strain (batch number AV1511) was obtained from the Guangxi University professor MeiLan Mo. This strain was propagated in ten-day-old SPF chicken embryos, which were provided by the Guangxi Veterinary Research Institute. Under sterile conditions, a 100μl sample of the IBV M41 strain was inoculated into the SPF chicken embryos. The survival of these embryos was monitored every 12 hours, and any that perished within the first 24 hours were excluded from the study. After 72 hours post-inoculation, allantoic fluid from the infected embryos was collected and stored at a temperature of -80°C. The median tissue culture infectious dose for SPF chicken embryo 50 percent Egg Infectious Dose (EID_50_) of the IBV M41 strain was determined using the Reed-Muench method. To prevent virus spread, all procedures were conducted in a Biosafety Level 2 (BSL-2) laboratory. Researchers wore appropriate personal protective equipment, including lab coats, gloves, and face masks. Inoculated embryos were placed in a dedicated, sealed incubator. Waste embryos and contaminated materials were immediately transferred to biohazard containers, and all equipment was disinfected with 75% ethanol after use. At the end of the experiment, all waste was autoclaved to ensure complete virus inactivation before disposal.

### 2.2 Reagents

The MXSG addition and reduction mixture consists of two blends, namely MXSG-mix1 (Licorice, Bitter Almond, Ephedra, Platycodon, Fritillaries) and MXSG-mix2 (Licorice, Bitter Almond, Ephedra, Platycodon, Stemona japonica, Rhizoma Belamcandae). These mixtures were prepared by blending the medicinal herbs in a 1:1 ratio and undergoing a triple decoction process. The Chinese medicinal materials were soaked in water for 30 minutes prior to boiling. Boiling was continued for an additional 15 minutes. The resulting liquid was then filtered through 3 layers of gauze. The remaining residue underwent another round of boiling using the same steps twice. The resulting poaching liquids from all three rounds were combined and concentrated to a consistency of 1g/ml using a vacuum pump and rotary evaporator at 0.08 MPa and 60°C. Finally, the concentrated poaching liquid was stored for future use. MXSG was obtained from Maybest Science and Technology Development Co., LTD., located in Hebei Province, China (Approval Number: 160036112; Production Batch Number is 20220403). According to the product description provided by the manufacturer, the composition includes ephedra, bitter almond, gypsum, and licorice with traditional Chinese medicine ingredients being present in approximately equal proportions. Additionally, it is stated that one milliliter of this positive drug is equivalent to 2.4 grams of the original crude drug.

### 2.3 Generate a modified formula of MXSG based on TCMICS V3.0

The clinical prescriptions for IBV were compiled from several databases to generate a modified formula of MXSG based on TCMICS V3.0, which is the Chinese Medicine Prescription database with 34,324 datasets (https://db.yaozh.com), the Chinese Patent Medicine Traditional Prescription cross-search database with 7,505 datasets, and another Chinese Patent Medicine Traditional Prescription cross-search database consisting of 33,837 traditional prescriptions (http://cpmtp.wangk.pro). Additionally, data was collected from the Drug Heritage Computing Platform that provided a dataset of 4,207 TCM formulations (TCMICS V3.0). All these datasets were relevant to the occurrence of chicken Infectious Bronchitis Virus and included in the statistical analysis. The prescription data was extracted and analyzed using TCMICS V3.0 data mining software to determine drug combination frequencies. A support threshold of ≥15% and confidence level of 0.5 were set to examine the association frequency between different drugs. The cluster analysis was then conducted by setting the cluster quantity to either 5 or 6, while restricting the number of TCM ingredients within each category to be between 5-6. By examining the compatibility patterns of TCM prescriptions and summarizing the combination rules within the database, a network topology analysis was conducted based on core drugs and original components of MXSG. This analysis led to the formulation of a modified MXSG mixture, which highlights the benefits and potential of integrating traditional Chinese medicine principles into veterinary practice.

### 2.4 Intraegg treatment

The nine-day-old SPF eggs were obtained from the Guangxi Veterinary Research Institute and placed in an incubator set at a temperature of 37.5°C for a 24-hour acclimation period. Subsequently, the viability of each egg was assessed using a flashlight. Ten-day-old chick embryos were then exposed to a 100-fold EID_50_ challenge dose, followed by uniform administration of TCM after 3 hours. A controlled blank group received an equivalent dose of normal saline. The study consisted of 8 groups: MXSG-mix1-H (0.36ml Chinese medicinal liquid, equivalent to 0.36g raw medicinal materials), MXSG-mix1-M (0.24ml Chinese medicinal liquid, equivalent to 0.24g raw medicinal materials), MXSG-mix2-H (0.36ml Chinese medicinal liquid, equivalent to 0.36g crude medicinal materials), MXSG-mix2-M (0.24ml Chinese medicinal liquid, equivalent to 0.24g crude medicinal materials), MXSG-H (0.15 ml Chinese medicinal liquid, equivalent to 0.36 g crude medicinal materials), MXSG-M (0.1 ml Chinese medicinal liquid, equivalent to 0.24 g crude medicinal materials), virus model group (IBV group), and blank group (NC group). Each experiment was replicated three times with 10 chicken embryos in each group. The presence of IBV in the embryos was determined by examining them for characteristic embryonic lesions associated with IBV, such as developmental retardation, coiling, and embryonic clubbing. This finding was confirmed by testing the allantoic fluid from chick embryos using the IBV RT-qPCR assay kit (NEQ13700-T, Harbin Sinosun Biotech Co., LTD., Harbin, China), following the manufacturer’s guidelines. Samples with a cycle threshold (Ct) of 38 or lower were considered positive for IBV infection. All eggs from each group were examined twice daily using a flashlight to detect any deceased embryos, which would then be placed in a refrigerator. After 7 days, various indicators of embryonic health, including the presence, quantity, and size of chorioallantoic membrane veins as well as embryo motility, were evaluated and compared to those of the control group.

At the conclusion of the experiment, all eggs from both the control and experimental groups were weighed. Then, their respective embryos were removed and washed. The weight of each embryo within each group was then measured to calculate the Embryo Index (EI) for all eggs using the following formula (Dhinakar Raj et al., 2004).


EI = [embryo weight (grams)/egg weight (gram)]×10000

### 2.5 Comparison of therapeutic effects in animal experiments

Two hundred and 41d chickens from Fufeng Group Co., LTD. (Nanning, China) were randomly divided into 8 groups, with 30 chickens per group. The groups included a control group without any treatment (NC group), a control group for challenge purposes (IBV group), MXSG-mix1-H group, MXSG-mix1-M group, MXSG-mix2-H group, MXSG-mix2-M group, MXSG-H positive drug group, and MXSG-M positive drug group. The chickens in the challenge control group were infected but did not receive any medication. The chickens in the blank control group remained uninfected and untreated throughout the entire 28-day experimental period. At 14 days of age, each chicken was weighed equally and simultaneously challenged. Medication (1ml of MXSG-M or 1.5ml of MXSG-H) was added to their drinking water for seven consecutive days, following the manufacturer’s dosage adjustment instructions. The dosage of MXSG-mix is​consistent with the corresponding raw materials of MXSG. After the challenge, 15 chickens were randomly selected from each group and subjected to clinical observation for signs of illness (**Table 1** clinical evaluation scoring criteria) as well as daily weight monitoring in the experimental group over a period of 14 days. The animal experiment was conducted in strict accordance with the guidelines set forth by the Animal Protection and Welfare Committee of Guangxi University (Approval Number: GXU-2023-0177), ensuring full compliance with ethical standards regarding animal ethics. During animal experiments, the infected chickens were housed in isolated facilities with restricted access. All handling of the animals was performed by trained personnel wearing protective gear, and the animal facilities were regularly disinfected. Any discarded bedding, excrement, and other potentially contaminated materials were treated as biohazard waste and disposed of following strict protocols.

**Table 1:** IBV experimental clinical evaluation score.

Check item	Clinical signs and symptoms	Points
Mental state	Lively and active, eyes bright	0
	The spirit is gloomy, the eyes are dull	2
	The spirit is weak, and the fear of cold gathers	4
Feeding status	Strong appetite, scramble for food	0
	Loss of appetite, leaving a lot of unfinished feed	2
	Loss of appetite and barely ate	4
Drinking state	Drinking water is normal	0
	Barely drinking water	4
Symptoms of head shaking	No head shaking	0
	Occasional shaking	2
	Frequent head shaking	4
Cough condition	No cough symptom	0
	Slight cough	2
	Cough frequently	4
Degree of tracheal rales	No tracheal crackles	0
	Slight rales in the trachea	2
	Severe rales in the trachea	4
Respiratory status	Eupnea	0
	Tachypnea	2
	Difficulty breathing, open mouth breathing	4

One day after discontinuation of medication (7d post-infection, 7 dpi), five chickens were selected from each group and euthanized. Under sterile conditions, the trachea was extracted and divided into sections: three rings from the upper section, four rings from the middle section, and three rings from the lower section for evaluation of the ciliary activity. The remaining middle section of the trachea was fixed in 10% formalin, embedded in paraffin at 60°C, and then sliced into 5-μm thick sections. The sections were dewaxed, dehydrated using a gradient of alcohol, and subsequently stained with H&E. After processing, the slices were dehydrated with gradient alcohol again, dewaxed, sealed with cover slips, and observed under a microscope. The ciliary swing scores were averaged across the five chickens, and a protection score was calculated (protection score = [1- average score of the treatment group/average score of the infection group] × 100) (Cook et al., 1999; Yan et al., 2016). Meanwhile, 1mL of blood was collected from the brachial vein and treated with an Ethylenediaminetetraacetic acid (EDTA) anticoagulant. Subsequently, routine blood parameters were determined using a full-automatic blood analyzer (TEK-VET3, TECOM, Jiangxi, China). These indexes included total white blood cell count (WBC), total red blood cell count (RBC), chicken hemoglobin content (HGB), hematocrit (HCT), mean corpuscular volume (MCV), mean corpuscular hemoglobin (MCH), mean corpuscular hemoglobin concentration (MCHC), platelet count (PLT), mean platelet volume (MPV), platelet distribution width (PDW), plateletcrit (PCT), percentage of intermediate cells (MID%), lymphocyte percentage (LYM%), and granulocyte percentage (GRAN%). On day 3 of medication (3 dpi), one day after cessation (7 dpi), and seven days after cessation (14 dpi), the spleen, thymus, and bursa of Fabricius were collected from five chickens in each group to calculate the immune organ index (Zhang et al., 2022). Nasal swab, trachea, lung, and kidney tissue samples were collected and subjected to viral nucleic acid extraction and reverse transcription using the StarSpin Virus DNA/RNA Kit, StarScript III All-in-one RT mix with gDNA Remover respectively (Genstar, Beijing, China). The IBV RT-qPCR detection kit (NEQ13700-T, Harbin Guosheng Biotechnology Co., Ltd., Harbin, China) was then employed for viral load detection in each organ. Serum samples were obtained for detecting six inflammatory factor indexes - IL-4, TNF-α, IFN-γ, IL-10, IL-6, and IL-1β - using an ELISA kit (Beyotime, Nanjing, China). Four oxidation indicators - MDA, SOD, T-AOC, XOD, and Nitric oxide - were measured using a biochemical reagent kit (Solarbio, Beijing, China). Total serum IBV specific antibodies were tested using an Indirect Enzyme-Linked Immunosorbent Assay (ELISA) kit (FZ067) provided by BioLayer. These tests were carried out in full accordance with the manufacturer’s instructions.

### 2.6 Component identification and network pharmacology analysis of MXSG-mix

#### 2.6.1 Identification of ingredients of modified and reduced mixture of MXSG-mix

The identification of the constituent elements in MXSG, a traditional Chinese medicine, as well as its additive and subtractive mixture, was accomplished using the Agilent 1290 Ultra Performance Liquid Chromatography (UPLC) system, coupled with the Thermo Fisher Q-Exactive Orbitrap Plus High Resolution Mass Spectrometer. Chromatographic conditions were set as follows: A Wates T3 column (21 mm×50 mm, 18 μm) was utilized at a column temperature of 50°C. The chromatographic solvents consisted of 0.1% formic acid in water (solvent A) and acetonitrile (solvent B). The flow rate was maintained at 0.3 mL/min. The gradient program was established as follows: 0–1 min at 2%, 1–18 min at 100%, 18–22 min at 100%, followed by a decrease to 2% from minute 22 to minute 22.1, and finally held constant until minute 25. The sample injection volume used was 5μL. Mass spectrometric conditions were set with a data-dependent acquisition (DDA) range spanning from 100 to 1000 m/z. The electrospray ionization (ESI) source conditions included an auxiliary gas flow rate of 16 Arb, a full MS resolution of 70000, a collision energy of 25 eV in the NCE model, an MS/MS resolution of 17500, and a spray voltage of -3.0 kV (negative) or 3.0 kV (positive). The system was configured with a dynamic exclusion time for 4 seconds, and the NCE was set to 10, 30, and 50 for data acquisition in the m/z range of 100-1000. The mass spectrometer operated in full scan mode. Xcalibur version 4.3 and MS-DIAL version 5.0.3 workstations were employed for data processing purposes. Compound identification involved comparing with reference materials, querying a locally constructed database, and confirming diagnostic ions. Compound identification relied on combining primary quasi-molecular ions with secondary mass spectrometry fragmentation rules.

#### 2.6.2 Acquisition and common target analysis of MXSG-mix components and targets

The study focused on analyzing the main components of the MXSG-mix, excluding any additional compounds. Initially, the first 50 compounds in both positive and negative states were examined to ensure data accuracy by eliminating redundant and unidentified samples. To identify potential targets, the study utilized the SwissTargetPrediction server (http://www.swisstargetprediction.ch/) and the STITCH tool for chemical interaction analysis (Szklarczyk et al., 2016). All identified drug targets were compiled into a comprehensive database, removing any duplicates. The SwissTargetPrediction database considers both 2D and 3D structural similarities of known small molecules for target prediction, while PharmMapper primarily identifies potential drug targets through reverse pharmacophore mapping (Gfeller et al., 2014). By integrating these methodologies, a rigorous and multifaceted approach to drug target identification within the field of veterinary science was accomplished. Additionally, the GeneCards (https://www.genecards.org/) and OMIM (https://omim.org/) databases were used in this study for identifying disease targets associated with the ‘chicken infectious bronchitis virus’. The identified genes from both databases were merged, removing any duplications, resulting in a comprehensive list of IBV-related targets. To identify common targets between the MXSG-mix and IBV, Venny 2.1 software (https://bioinfogp.cnb.cic.es/tools/venny/) was employed. Furthermore, Cytoscape_3.8.0 software was used to construct a visual network diagram illustrating the relationship among the active ingredients of MXSG-mix, their respective targets, and the IBV targets. For protein interaction information related to overlapping targets, the STRING database (https://www.string-db.org/) was utilized. The species parameter was set as ‘Gallus’, and a confidence level above 0.04 was chosen. This approach facilitated identifying key target proteins while providing valuable insights.

#### 2.6.3 GO\KEGG analysis of IBV treated with MXSG-mix

The impact of MXSG-mix on key target proteins related to IBV was analyzed using the cluster Profiler package in R software. A corrected p-value threshold of less than 0.05 was employed as the screening criterion. The genes were categorized based on the similarity among their members (Last updated: October 30, 2023). This approach allowed for a comprehensive investigation of the actions and pathways of the intersecting targets, providing insights into the therapeutic targets, providing valuable insights into the therapeutic targets and signaling pathways of MXSG-mix in IBV treatment (Yu et al., 2012; Luo and Brouwer, 2013).

The Hubba gene module in Cytoscape_v3.7.1 software was used for assessing intersecting genes, with the aim of facilitating subsequent data interpretation and analysis. To analyze network topology, the maximum neighborhood component (MNC) was employed as a metric to measure the average distance between one node and all other nodes. The significance of these parameters lies in their ability to demonstrate the topological prominence of individual nodes within the network.

### 2.7 Real-time quantitative polymerase chain reaction

After a 24-hour drug discontinuation period, five chickens from each group were selected for euthanasia at 7 dpi. Trachea and spleen extractions were then performed to measure the expression levels of the top three differentially expressed genes identified by the MNC algorithm, as described in Method 2.6.4. RNA extraction from the trachea and spleen specimens was carried out using Sevier’s Animal Tissue/Cell Total RNA Extraction kit (Hubei, China), following the manufacturer’s guidelines. The cDNA samples were synthesized using the StarScript III All-in-one RT mix with gDNA Remover reverse transcription kit (Genstar Corporation, Beijing, China). Real-time RT-qPCR was performed using the 2× Real Star Green Fast mixture system (GenStar, China), following the manufacturer’s protocol. Gene normalization was achieved through utilization of reference genes. The primer sequences for both internal reference gene and the target genes used in RT-qPCR can be found in Additional file 1. Primer specificity was confirmed through testing, and all RT-qPCR experiments were performed in triplicate to analyze the expression levels of the candidate genes.

To investigate the crucial compounds that influence the primary target proteins, data was collected from drug-disease target outcomes. Subsequently, the relevant compounds were batch imported into the discovery platform for molecular docking, which provided docking scores. A higher absolute value of the docking score indicates a greater likelihood of compound-target binding. Typically, results with an absolute value of 5 or above suggest potential binding possibilities. The obtained results were visualized using R language to create a heatmap for visualization purposes. Additionally, Python software was used to perform molecular docking display based on the highest score identified in the heatmap.

### 2.8 Statistical analysis

The experimental units for this study were defined as sets of replications; each set consisted of either fifteen (n = 15) or five (n = 5) replications treated as single units, respectively. All collected data underwent statistical analysis using GraphPad Prism 9 software. Key variables assessed included viral load, immune organ index, titer of IBV-specific antibodies, and cytokine concentrations. These variables were statistically examined through one-way analysis of variance (ANOVA) to identify any significant differences. Subsequently, a *post hoc* analysis was performed using the Tukey test to further discern significant variances between the different treatment groups. The level of significance was set at P < 0.05, with outcomes below this threshold considered statistically significant. Outcomes with a P-value less than 0.01 were deemed highly significant within this rigorous and thorough statistical framework. In academic figures, symbols like * , ** , *** , **** generally represent statistical significance: * denotes p < 0.05, ** p < 0.01, *** p < 0.001, and **** p < 0.0001, indicating the probability that results occurred by chance, with smaller symbols corresponding to higher significance.

## 3 Results

### 3.1 TCMICS V3.0 was used to generate MXSG increasing and decreasing mixture

**Figure 1:**
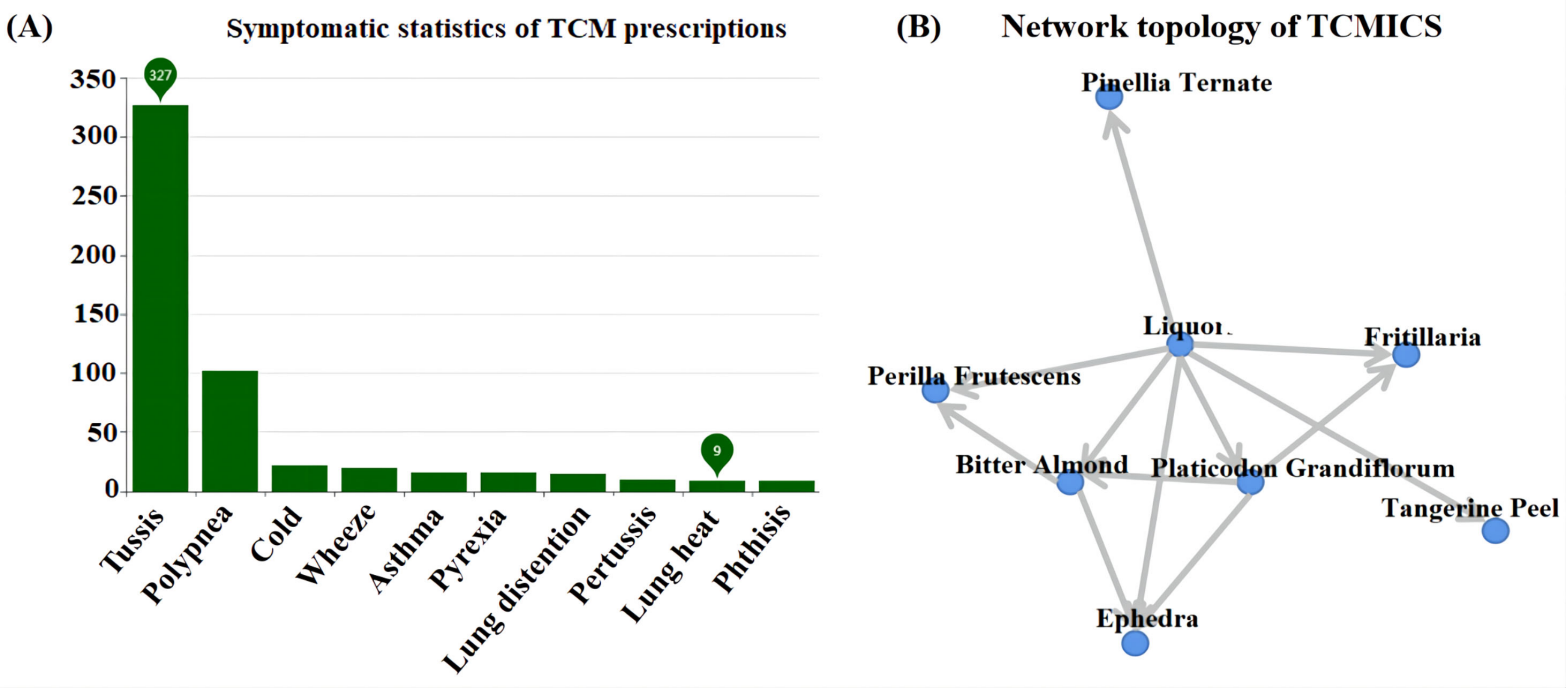
**(A)** Database of chicken infectious bronchitis; **(B)** TCMICS association rule network topology. The number of prescriptions represents a collection of prescriptions with similar drug compositions, where each prescription is considered as a prescription-like element.

A comprehensive examination was conducted on a total of 353 distinct prescriptions intended for the treatment of cough, encompassing 361 unique drugs. This led to the construction of a targeted database for the treatment of IBV (**Figure 1A**) and a network topology illustrating the association rules between different prescriptions (**Figure 1B**). Subsequently, a cluster analysis was performed on these 353 prescriptions and 361 Chinese herbs, resulting in the creation of two new prescriptions, MXSG-mix1 and MXSG-mix2, both belonging to the core class of the MXSG group (**Table 2**). Compared to the traditional MSXG, MXSG-mix1 is an improved version that excludes gypsum while including Platycodon and Fritillaries, with the database revealing similarities in composition with other formulas numbering at least 127. Meanwhile, MXSG-mix2 also eliminates gypsum but introduces platycodon radix, Stemona japonica, and Rhizoma Belamcandae, with comparable formulas found in the database amounting to at least 62. The cost of these optimized drug is similar to that of Maxing Shigan Decoction, a high-quality and cost-effective option. The dosage utilized aligns with the standard dosage recommended for Maxing Shigan Decoction, a common used treatment in Chinese poultry farms for addressing respiratory issues in chickens.

**Table 2:** Analysis of core medication combinations.

Number	Drug pairs and combinations	Quantity of prescription
MXSG	Ephedra, bitter almond, plaster, and licorice	–
MXSG-mix1	Licorice, Bitter Almond, Ephedra, Platycodon, Fritillaries	127
MXSG-mix2	Licorice, Bitter Almond, Ephedra, Platycodon, Stemona japonica, Rhizoma Belamcandae	62

### 3.2 Comparison of *in vitro* treatment effects based on chicken embryos

**Figure 2:**
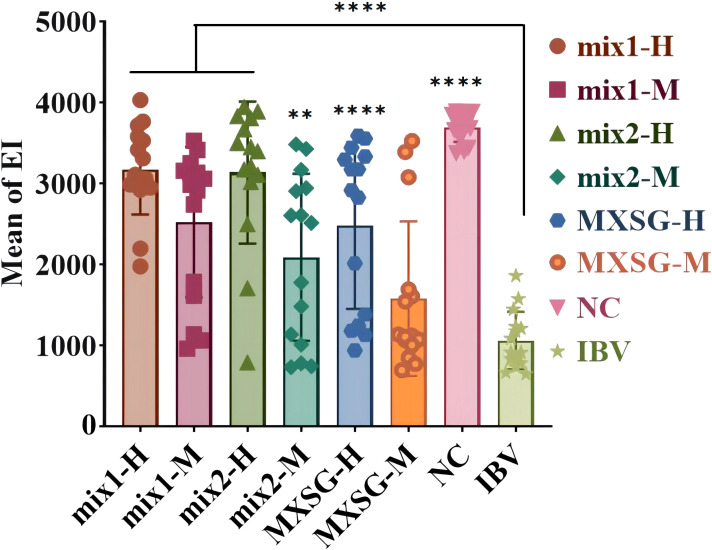
The inhibitory effect of MXSG or MXSG-mix on IBV (M41) *in vivo*.

The SPF chicken embryos infected with the IBV-M41 strain did not exhibit any egg fatalities within the initial 24-hour period. After an incubation period of 5 days, the embryos were dissected to assess the extent of lesions. The EID_50_ was determined using the Reed-Muench method and found to be 10^-6.16^/1mL. **Figure 2** illustrates the injection of a dose containing 100 EID_50_ units of the IBV M41 strain into the chicken embryos, resulting in substandard overall development, reduced sizes, and deformities. All these embryos perished during the observed week, with an EI measuring at 1064.170 ± 354.958. In contrast, the control group that did not receive any injection exhibited a survival rate of 100% and an EI measuring at 3692.133 ± 176.685. Thus, it is evident that the IBV-M41 injection was solely responsible for the significant reduction in EI. When compared to the IBV group, the traditional Chinese medicine treatment groups demonstrated a notable increase in EI. Among these groups, the MXSG-mix1-H treatment proved to be the most effective in mitigating impact of IBV-M41 on embryos, achieving an EI of 3175.458 ± 555.279, which was significantly higher than that of the MXSG-H group at 2482.197 ± 1028.701. Furthermore, MXSG-mix1-H showed minimal toxicity towards the chicken embryos by reducing their mortality rate and improving their dysplasia situation. Therefore, it can be concluded that MXSG-mix1-H serves as an efficacious drug treatment for embryos infected with the IBV M41 strain.

### 3.3 *In vivo* antiviral effect of MXSG-mix against avian infectious bronchitis virus

#### 3.3.1 Clinical sign score and daily weight gain

**Figure 3:**
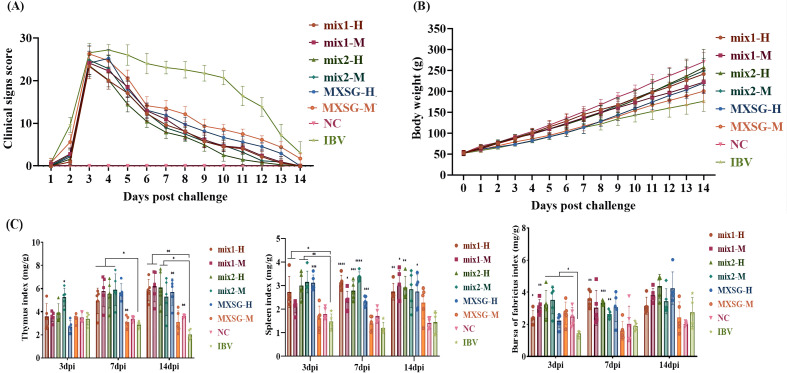
**(A)** Clinical evaluation; **(B)** Average daily weight gain. The research subjects were the same batch of chickens, 15 in each group; **(C)** Immune organ index: Thymus, Spleen, French bursa.

The clinical symptoms and daily weight gain of chicks infected with IBV were observed. Following IBV infection, a significant decrease in symptom scores was observed in all TCM treatment groups, while the virus group consistently showed higher symptom scores. A notable decline in symptom scores was observed at the 3 dpi mark in all drug groups, indicating a critical turning point. Among these groups, the MXSG-mix2-H group exhibited the lowest symptom scores at 5 dpi. By the 14 dpi mark, all TCM treatment groups, except the MXSG-M group, had reduced symptom scores to zero (**Figure 3A**). In terms of growth performance, both dosage levels of the MXSG-mix2 group outperformed other treatment groups. The growth curve of this group closely resembled that of the control group (**Figure 3B**). Analyzing the trend of curve change leads to concluding that the MXSG-mix2-H group demonstrated superior clinical performance in combating the effects of IBV infection.

#### 3.3.2 Immune organ index

A comprehensive analysis was conducted at specific intervals of 3 dpi, 7 dpi, and 14 dpi to evaluate the immune organ index, with a focus on critical organs such as the spleen, thymus, and bursa of Fabricius. Five chickens were selected from each group, and their respective organ weights were measured to calculate the relative organ weight (**Figure 3C**). At 3 dpi, there was a noticeable increase in the thymus index of the MXSG-mix2-M group, indicating a significant difference compared to the challenge group. By 7 dpi, most of the Chinese medicine treatment groups showed clear distinctions from the challenge group, except for the MXSG-M group. The MXSG-H group demonstrated a pronounced discrepancy that persisted until the 14 dpi mark. Consistently throughout the experiment, the treated group displayed higher spleen indices compared to those of the challenge group. Notably, no discernible distinction was observed between the MXSG-mix2-M and blank groups within this timeframe. Regarding the bursal index, a significant decrease was recorded in the IBV group at 3 dpi. However, each drug treatment group showed a significant increase in the bursal index compared to the challenge control group. By 7 dpi, both the MXSG-mix1-H group and the MXSG-mix2 group maintained a consistently high bursa index. By 14 dpi, there were no longer any statistically significant differences between the groups.

#### 3.3.3 Analysis of tracheal histopathology and cilia swing score

**Figure 4:**
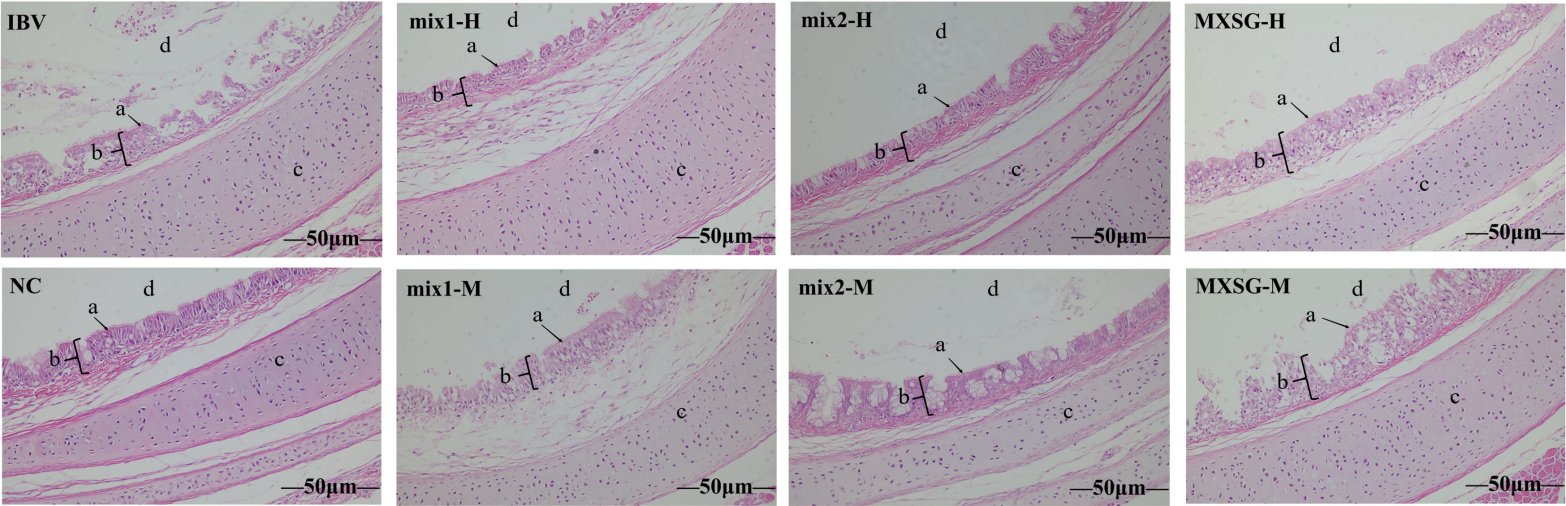
HE stains demonstrates the beneficial impact of traditional Chinese medicine treatment on tracheal histopathological changes induced by IBV (magnification × 200). In the image, ‘a’ denotes cilia, ‘b’ represents tracheal mucosa, ‘c’ signifies tracheal cartilage, and ‘d’ represents tracheal lumen.

At 7 dpi, five chickens from each group were euthanized for further examination. Tracheal rings were extracted and evaluated under a light microscope to assess the health of cilia. Observations revealed that the challenge group exhibited excessive tracheal mucus, impaired cilia movement, and severe cases showed loss of cilia. The average viability score for tracheal cilia in this group was 38.5, indicating an absence of protection. In contrast, the MXSG-mix groups showed a tracheal protection rate exceeding 60%, with the MXSG-mix2-H group achieving the highest rate at 70.62% (**Table 3**). On the other hand, the MXSG group’s protection rate remained below 60%. Histopathological examination corroborated these findings (**Figure 4**). In the negative control (NC) group, the chicks’ tracheas appeared normal without any pathological changes. However, the IBV group showed severe tracheal damage, characterized by desquamation of cilia, extensive infiltration of inflammatory cells in the submucosa, as well as a predominance of mononuclear cells and lymphocytes in the submucosa. In contrast, the treatment group exhibited reduced tracheal lesions. The MXSG-mix2-H group displayed the mildest pathological changes with minor cilia shedding, gland degeneration, and congestion. The MXSG-H, MXSG-M, and MXSG-mix1-M groups showed significant infiltration of monocytes and lymphocytes along with hemorrhage, glandular hyperplasia, as well as mucosal monocyte and lymphocyte infiltration. The therapeutic efficacy of the remaining drug groups fell between that observed for the MXSG-mix2-H and MXSG-mix1-M groups.

**Table 3:** The ciliary score results.

Group	Ciliary score of the trachea						
	Individual score					Average score	Protective scoring
MXSG-mix1-H	13	12	13	10	10	11.6	67.23%
MXSG-mix1-M	14	13	16	12	15	14	60.45%
MXSG-mix2-H	13	11	11	7	10	10.4	70.62%
MXSG-mix2-M	17	13	13	14	12	13.8	61.025
MXSG-H	16	15	16	16	13	15.2	57.06%
MXSG-M	19	12	18	18	20	17.4	50.85%
NC	0	0	0	0	0	0	100.00%
IBV	36	32	32	39	38	35.4	0.00%

#### 3.3.4 Blood routine test

At 7 dpi, venous blood samples were collected from five chickens in each treatment group for a complete blood count (CBC) test. The results revealed that the influence of various TCM compounds on the hematological parameters of chickens varied depending on the collection time. Detailed findings can be found in **Table 4**. When comparing each treatment group with the challenge group, it was observed that the MXSG-mix1 treatment had a significant impact on certain blood parameters including WBC at 3 dpi, HCT at 14 dpi, MCV at 3 dpi, and PDW in the M group at 3 dpi. Additionally, it significantly affected PCT in the H group at 3 dpi, as well as LYM% and GRAN% in the H group at both 3 dpi and 7 dpi. The MXSG-mix2 treatment significantly influenced HCT in the M group at 14 dpi, MCV and PDW in the M group at 3 dpi, and MID% in both the M group at 3 dpi and H group at 7 dpi. Furthermore, it notably affected GRAN% in the H group at 7 dpi. The MXSG-H treatment was found to significantly affect HCT, MCV, MCH, PLT, and MPV levels at 14 dpi as well as MID% and LYM at 7 dpi. In summary, TCM treatment began to affect most blood indices by 3 dpi with certain effects persisting up to a week after the cessation of treatment. The overall improvement in health conditions was comparable to that of the control group.

**Table 4:** Routine blood test.

Ciliary Score Results	mix1-H	mix1-M	mix2-H	mix2-M	MXSG-H	MXSG-M	NC	IBV
WBC 3 dpi	216.50 ± 9.52***	212.88 ± 16.22*	203.44 ± 40.81	181.42 ± 36.00	188.70 ± 24.05	190.08 ± 34.95	184.56 ± 8.30	175.44 ± 9.09
WBC 7 dpi	188.86 ± 10.59	200.42 ± 20.12	198.14 ± 7.85	187.50 ± 19.30	179.72 ± 8.78	192.60 ± 8.69	189.02 ± 3.62	187.46 ± 8.72
WBC 14 dpi	183.42 ± 20.77	195.70 ± 8.20	186.26 ± 14.71	168.68 ± 15.91	176.84 ± 9.89	183.32 ± 29.43	191.58 ± 2.87	195.80 ± 15.80
RBC 3 dpi	3.25 ± 0.09	3.26 ± 0.21	2.80 ± 0.50	3.18 ± 0.25	3.16 ± 0.43	3.11 ± 0.20	2.84 ± 0.13*	3.35 ± 0.24
RBC 7 dpi	3.01 ± 0.21	2.84 ± 0.33	2.74 ± 0.35	3.16 ± 0.16	3.04 ± 0.18	3.33 ± 0.18	2.87 ± 0.14*	3.22 ± 0.13
RBC 14 dpi	2.86 ± 0.27	2.74 ± 0.25	2.74 ± 0.26	2.96 ± 0.16	2.81 ± 0.14	2.79 ± 0.16	2.81 ± 0.12	2.72 ± 0.40
HGB 3 dpi	100.40 ± 9.61	97.60 ± 9.66	91.60 ± 16.13	88.80 ± 16.81	90.80 ± 4.97	92.60 ± 10.85	67.80 ± 12.32**	99.40 ± 8.11
HGB 7 dpi	94.40 ± 16.70	81.60 ± 13.94	75.60 ± 28.09	85.40 ± 10.50	81.60 ± 10.43	105.60 ± 9.29	72.80 ± 6.83*	104.60 ± 17.24
HGB 14 dpi	93.20 ± 27.42	84.80 ± 5.72	79.80 ± 8.53	76.00 ± 5.10	81.20 ± 6.46	92.00 ± 22.84	77.40 ± 8.56	90.80 ± 16.99
HCT 3 dpi	31.20 ± 2.25	30.30 ± 3.13	28.88 ± 6.71	26.86 ± 5.09	26.04 ± 3.35	27.84 ± 2.95	23.16 ± 2.24*	30.56 ± 2.88
HCT 7 dpi	33.34 ± 8.22	31.32 ± 1.75	30.36 ± 2.62	28.16 ± 1.95	30.04 ± 1.72	34.84 ± 3.44	23.82 ± 2.19*	33.00 ± 4.55
HCT 14 dpi	23.14 ± 1.69***	25.00 ± 2.34*	28.28 ± 1.50	26.56 ± 1.53*	26.30 ± 2.33*	29.50 ± 3.26	22.86 ± 2.35**	30.74 ± 1.72
MCV 3 dpi	138.64 ± 5.29*	137.86 ± 4.67*	132.10 ± 9.72	136.34 ± 2.95**	135.82 ± 7.91	134.92 ± 5.31	130.20 ± 5.29	128.28 ± 2.54
MCV 7 dpi	137.34 ± 1.67	134.36 ± 3.15	133.96 ± 4.96	132.64 ± 3.77	130.04 ± 2.75	131.82 ± 3.66	127.94 ± 2.29	131.72 ± 3.74
MCV 14 dpi	136.66 ± 2.58	130.56 ± 4.94	130.44 ± 3.36	135.92 ± 4.32	137.08 ± 1.01*	132.64 ± 2.08	130.56 ± 1.16	131.48 ± 2.58
MCH 3 dpi	44.50 ± 3.08	44.04 ± 1.47	43.80 ± 4.14	44.90 ± 1.20	45.28 ± 4.39	43.10 ± 3.05	39.80 ± 2.83	42.52 ± 1.40
MCH 7 dpi	46.40 ± 3.57	42.78 ± 2.09	43.32 ± 1.63	42.00 ± 1.59	41.08 ± 1.99	44.04 ± 3.44	38.28 ± 1.26*	42.02 ± 1.41
MCH 14 dpi	46.36 ± 1.83	43.58 ± 2.02	42.84 ± 1.26	46.52 ± 1.82	45.74 ± 0.49*	44.94 ± 0.85	38.66 ± 1.65**	43.98 ± 0.76
MCHC 3 dpi	320.80 ± 10.73	319.60 ± 6.73	331.80 ± 17.30	330.20 ± 3.70	333.20 ± 13.83	320.20 ± 15.58	309.40 ± 12.74	330.40 ± 6.35
MCHC 7 dpi	344.40 ± 30.14	317.80 ± 16.84	324.20 ± 8.35	317.40 ± 3.44	314.20 ± 11.69	337.60 ± 11.28	308.40 ± 7.16	314.80 ± 13.08
MCHC 14 dpi	339.60 ± 8.59	334.20 ± 5.89	328.80 ± 9.18	341.80 ± 9.98	334.60 ± 3.97	338.80 ± 10.38	310.60 ± 4.16***	334.80 ± 5.26
PLT 3 dpi	65.20 ± 16.69	43.20 ± 14.75	49.00 ± 11.85	51.60 ± 30.11	53.60 ± 8.50	38.60 ± 22.55	68.00 ± 2.55***	38.60 ± 6.07
PLT 7 dpi	31.00 ± 5.66	28.40 ± 0.89	37.80 ± 17.47	36.00 ± 19.75	35.80 ± 9.31	43.60 ± 24.34	67.20 ± 4.55**	36.60 ± 10.26
PLT 14 dpi	60.60 ± 19.97	58.40 ± 18.47	41.80 ± 3.70	41.20 ± 6.98	42.00 ± 7.68	39.80 ± 5.54	68.40 ± 3.05*	39.00 ± 14.78
MPV 3 dpi	6.18 ± 0.47	6.80 ± 0.69	6.44 ± 0.27	6.32 ± 0.37	6.58 ± 0.19*	6.34 ± 0.71	5.34 ± 0.17*	6.00 ± 0.29
MPV 7 dpi	6.16 ± 0.63	6.38 ± 0.26	5.84 ± 0.45	6.04 ± 0.11	6.12 ± 0.27	6.00 ± 0.39	5.30 ± 0.26	6.02 ± 0.87
MPV 14 dpi	5.58 ± 0.76	5.48 ± 0.69	5.56 ± 0.48	5.48 ± 0.89	5.78 ± 0.56	5.78 ± 0.80	5.50 ± 0.32	6.22 ± 0.37
PDW 3 dpi	18.60 ± 0.55	18.98 ± 0.41*	17.14 ± 4.16	18.60 ± 0.42*	18.98 ± 0.29	18.64 ± 0.83	18.98 ± 0.41*	18.00 ± 0.38
PDW 7 dpi	18.28 ± 1.42	18.18 ± 1.40	18.82 ± 0.58	17.96 ± 1.73	17.92 ± 1.65	18.46 ± 1.37	18.74 ± 0.17	18.62 ± 1.07
PDW 14 dpi	18.66 ± 0.68	18.82 ± 0.44	18.44 ± 0.89	18.34 ± 0.09	18.60 ± 0.44	17.96 ± 0.11	18.64 ± 0.48	18.04 ± 0.81
PCT 3 dpi	0.04 ± 0.01*	0.03 ± 0.02	0.04 ± 0.02	0.03 ± 0.02	0.04 ± 0.02	0.02 ± 0.02	0.06 ± 0.01***	0.02 ± 0.00
PCT 7 dpi	0.02 ± 0.01	0.01 ± 0.01	0.02 ± 0.01	0.02 ± 0.02	0.02 ± 0.01	0.02 ± 0.02	0.06 ± 0.01*	0.03 ± 0.01
PCT 14 dpi	0.04 ± 0.02	0.05 ± 0.02	0.04 ± 0.01	0.03 ± 0.01	0.03 ± 0.01	0.03 ± 0.01	0.06 ± 0.00**	0.02 ± 0.01
MID% 3 dpi	13.83 ± 1.46	13.25 ± 0.93	12.81 ± 0.45	11.47 ± 0.93*	12.59 ± 0.73	13.14 ± 1.17	7.82 ± 0.15**	14.03 ± 1.19
MID% 7 dpi	11.57 ± 1.21	11.85 ± 1.22	10.18 ± 0.76*	11.28 ± 1.56	10.51 ± 1.29	11.12 ± 0.72	7.54 ± 0.19**	12.71 ± 1.39
MID% 14 dpi	8.83 ± 0.74	10.17 ± 1.59	8.74 ± 0.96	9.41 ± 1.30	8.74 ± 0.41*	9.86 ± 1.00	7.82 ± 0.16**	10.21 ± 0.60
LYM% 3 dpi	86.21 ± 2.07*	83.99 ± 4.72	83.53 ± 2.40	85.43 ± 1.80	84.52 ± 4.00	84.02 ± 4.88	86.70 ± 2.61*	80.04 ± 3.13
LYM% 7 dpi	79.86 ± 3.88*	77.31 ± 2.69	78.68 ± 7.15	78.97 ± 4.82	79.64 ± 3.71*	75.26 ± 3.82	86.58 ± 2.48***	72.38 ± 2.47
LYM% 14 dpi	83.88 ± 1.91	83.97 ± 3.65	84.06 ± 1.87	82.61 ± 1.17	83.44 ± 1.93	83.68 ± 3.05	84.78 ± 1.38	81.76 ± 5.31
GRAN% 3 dpi	12.67 ± 0.88**	13.20 ± 0.43*	13.15 ± 1.49	14.40 ± 1.08	13.63 ± 1.67	13.73 ± 1.23	9.81 ± 0.55****	15.24 ± 0.87
GRAN% 7 dpi	13.77 ± 1.48	13.36 ± 1.48	12.45 ± 0.84*	11.98 ± 1.44	14.99 ± 1.21	14.42 ± 1.47	9.68 ± 0.24***	14.39 ± 0.83
GRAN% 14 dpi	12.31 ± 0.48	12.33 ± 0.98	11.60 ± 0.50	12.15 ± 1.04	11.56 ± 0.61	11.99 ± 0.89	9.64 ± 0.30*	11.53 ± 0.82

#### 3.3.5 Viral load

**Figure 5:**
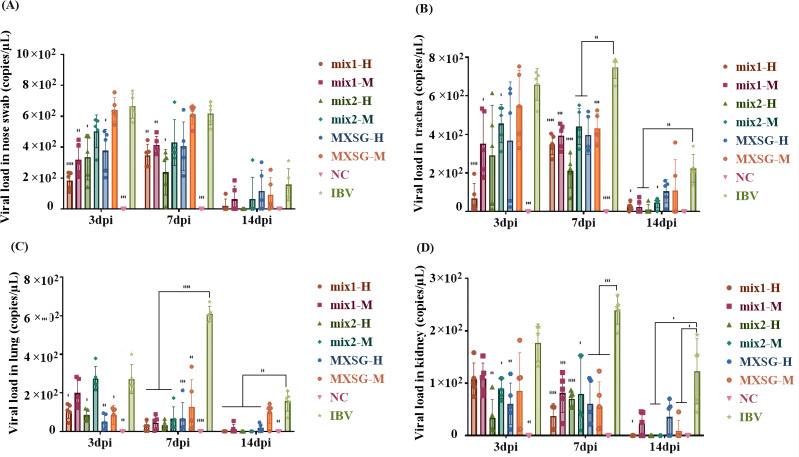
Viral load: **(A)** Nasal swab; **(B)** Trachea; **(C)** Lung; **(D)** Kidney.

In order to assess the efficacy of each TCM group in suppressing IBV, the levels of viral RNA were measured in nasal swabs, trachea, lung, and kidney across different groups (**Figure 5**). Real-time qPCR was employed at three time points: 3 dpi, 7 dpi, and 14 dpi. No detectable virus was observed in the trachea or kidney of the NC chicken group. However, compared to the drug-treated group, significantly higher expression levels of IBV RNA were detected in both the trachea and kidney of the IBV-infected group. Moreover, a higher viral load was found in nasal swabs and trachea as opposed to lung and kidney. The TCM treatment group was able to significantly reduce viral invasion into the lungs at 7 dpi. At 3 dpi, the MXSG-mix1-H group showed superior antiviral capacity in the respiratory tract compared to other groups. Whereas, at 7 dpi, the MXSG-mix2-H group displayed enhanced antiviral capacity in the respiratory tract. Throughout the entire experimental period, except for lung detection indices at 3 dpi, the MXSG-mix-H group consistently exhibited a stronger antiviral effect than the MXSG-H group. By 14 dpi, the viral load started to decrease in all groups, but this decrease was more pronounced in the samples treated with traditional Chinese medicine. Notably, the MXSG-mix2-H group recorded the lowest viral load levels approaching those of blank controls across all organs.

#### 3.3.6 Inflammatory factors

**Figure 6:**
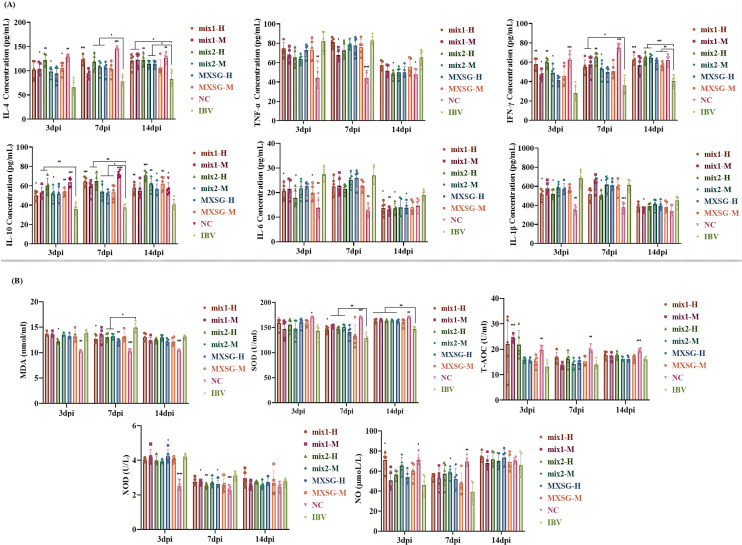
**(A)** Inflammatory factor markers; **(B)** Oxidation factor index.

The experiment investigated the effects of MXSG-mix and MXSG on inflammatory responses in chickens infected with IBV. The study focused on quantifying the expression levels of various cytokines, including anti-inflammatory cytokines such as IL-4, IL-10, and type II interferon IFN-γ, as well as pro-inflammatory cytokines like tumor necrosis factor TNF-α, IL-6, and IL-1β (as shown in **Figure 6A**). In comparison to the NC group, the IBV-infected group exhibited a downregulation in the production of IL-4, IFN-γ, and IL-10, along with an upregulation in TNF-α, IL-6, and IL-1β. At 3 dpi, significant differences were observed between the MXSG-mix2-H group and the IBV-infected group for all indices except TNF-α. Notably, this group demonstrated a dose-dependent anti-inflammatory response surpassing that of other TCM formulas tested. By 7 dpi, the TCM groups still differed significantly from the IBV-infected group for IL-4, IFN-γ, and IL-10 levels. However, the only notable difference was observed between the MXSG-mix1-M and IBV groups for TNF-α levels. The MXSG-mix1-H group exhibited superior anti-inflammatory effects compared to the other TCM formulas. The only significant differences in IL-1β were observed between the MXSG-mix2-H and IBV groups. By the fourteenth day 14 dpi, the MXSG-mix1-M group demonstrated a more pronounced anti-inflammatory response than the other TCM formulas, as evidenced by the significant differences in IL-4, IFN-γ, IL-10, and IL-6 levels between the TCM and IBV groups.

#### 3.3.7 Oxidation factors

In this study, the impact of MXSG-mix and MXSG on oxidative stress markers in IBV-infected chickens was investigated, including MDA, SOD, T-AOC, XOD, and NO. As depicted in **Figure 6B**, the levels of SOD, T-AOC, and NO were significantly lower in the experimental groups compared to the NC group after IBV infection. However, treatment with MXSG-mix1-H led to a significant increase in NO levels at 3 dpi, while MXSG-mix1-M treatment significantly boosted T-AOC levels at the same time-point. Moreover, by 7 dpi, all four MXSG-mix treated groups showed a substantial elevation in SOD levels. Conversely, the levels of XOD and MDA decreased in the experimental groups compared to the NC group after IBV infection. However, administration of MXSG-mix2-H effectively attenuated the decline in XOD and MDA levels at 7 dpi with a significant difference observed from the IBV group. By 14 dpi, there were no significant differences observed across all experimental groups regarding XOD and NO levels, while MDA, T-AOC, and SOD levels resembled those of the blank group. Overall, the performance of MXSG-mix group surpassed that of the positive control drug in terms of the five antioxidant indices.

#### 3.3.8 Antibody level

**Figure 7:**
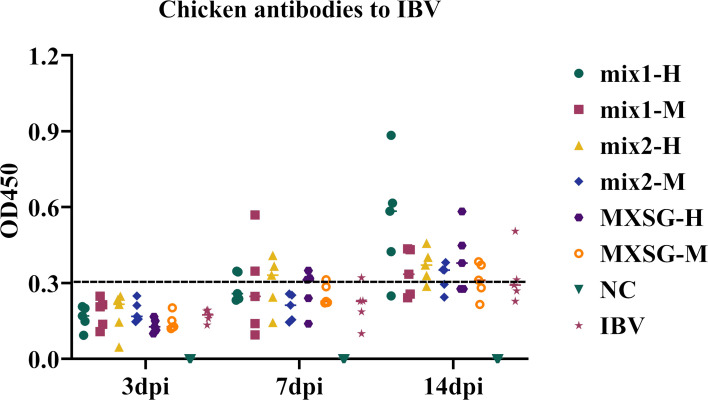
Antibody levels.

The potential of MXSG and MXSG-mix in enhancing humoral immune responses in poultry was investigated by quantifying antibody OD values using ELISA. As shown in **Figure 7**, following the challenge, both the IBV and drug groups exhibited significantly increased antibody concentrations, surpassing those observed in the NC group. At 3 dpi, the IBV group displayed higher antibody titers compared to the NC group, but this difference did not reach statistical significance when compared to the treated group. By 7 dpi, the MXSG-mix2-H group demonstrated higher levels of antibodies than the IBV group, with three subjects showing noticeable antibody production (OD_450_ > 0.3). At the 14 dpi mark, the MXSG-mix1-H group had the highest antibody content, and a greater number of subjects in the MXSG-mix2-H group showed significant antibody production compared to those in the MXSG group. These findings suggest that MXSG-mix enhances the humoral immune response dose-dependently and outperforms both MXSG and IBV groups.

### 3.4 Identification of components and network pharmacology analysis

#### 3.4.1 Identification of ingredients in MXSG-mix

**Figure 8:**
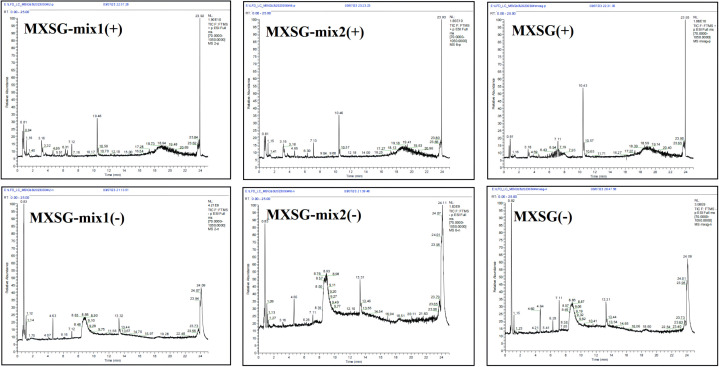
Base Peak Intensity of MXSG and MXSG-mix.

Following the administration and comprehensive study of MXSG and MXSG-mix testing solutions, Base Peak Intensity (BPI) mappings were generated in both positive and negative ion modes, as depicted in **Figure 8**. The primary mass spectra in these ion modes facilitated the identification of molecular ion peaks, enabling an in-depth analysis of secondary fragment data. This analysis led to a partial structural inference of the compound under investigation. The peak attributions were established by comparing the total ion current maps of the mixture and individual medicinal substances. Through the utilization of the HMDB database, the compounds that are most likely to be present were deduced. In this analysis, MXSG-mix1 and MXSG-mix2 revealed the detection of 1912 and 1138 compounds respectively. These compounds were derived from the primary components extracted from MXSG-mix. A thorough analysis was conducted on the top 50 compounds in each direction, followed by eliminating unknown samples and redundant data, resulting in a final total of 90 identified compounds in MXSG-mix1 and 86 compounds in MXSG-mix2, as presented in Additional file 2.

#### 3.4.2 Construction of MXSG-mix chemical targets-IBV disease target network

**Figure 9:**
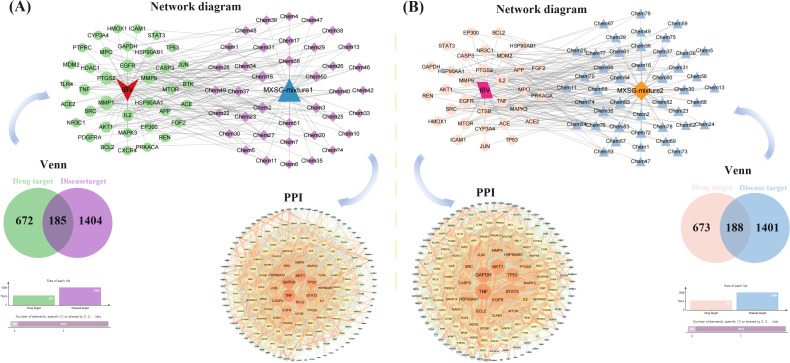
The construction of a drug target protein-IBV differential protein interaction network. **(A)** shows the number of potential targets and target interactions of MXSG-mix1 against IBV disease; **(B)** presents the same for MXSG-mix2. In both sub - figures, the circle represents the intersection of MXSG-mix and IBV target proteins. The size and darkness of the circle indicate the degree value, with larger and darker circles meaning higher values. The lines represent interactions between target proteins, where thicker and darker lines signify stronger interactions.

The network pharmacology analysis encompassed a total of 41 compounds from MXSG-mix1 and 57 compounds from MXSG-mix2. Duplicate target proteins were excluded, resulting in the identification of 857 drug target proteins for MXSG-mix1 and 861 drug target proteins for MXSG-mix2. These target proteins were used to create the self-built database for MXSG-mix (Additional file 3). To identify chicken IBV-related target proteins, a search was conducted in the GeneCards data library and OMIM database using the keyword ‘infectious bronchitis virus’. The obtained genes from these databases were merged and duplicates were eliminated, leading to the identification of IBV-related target proteins (Additional file 4). The Venn diagram was utilized to determine both key target proteins of MXSG-mix and IBV-related target proteins. A total of 185 genes from MXSG-mix1 and 188 genes from MXSG-mix2 were found to be active components in treating IBV. The potential key target proteins of MXSG-mix in the treatment of IBV were further investigated using the STRING database (Additional file 5-6). Subsequently, Cytoscape was employed to construct a protein-protein interaction (PPI) network for visualization purposes. Circular nodes were used to represent the target proteins, with their size and brightness indicating their degree and mediation centrality, respectively. The strength of interactions between the target proteins was depicted by line thickness and color depth of the edges, where brighter and thicker lines denoted closer interactions. Degrees of targets ranged from light to dark orange, with larger nodes representing higher degrees and thicker edges indicating stronger interactions. The resulting PPI network is illustrated in **Figure 9**.

#### 3.4.3 Key targets and pathway analysis

**Figure 10:**
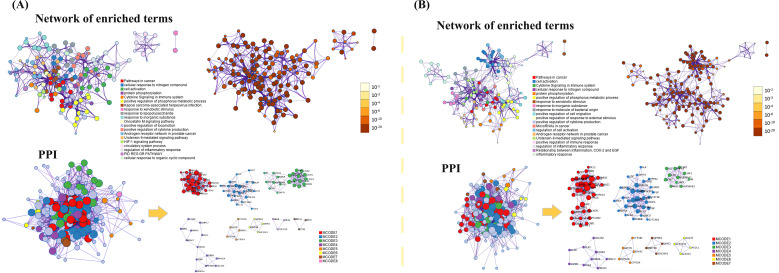
The results of functional enrichment analysis for key targets. **(A)** MXSG-mix1, **(B)** MXSG-mix2. The network diagram illustrates the enriched terms, with circles representing cluster IDs colored accordingly. Nodes with the same cluster ID are typically close to each other. On the right side, a network diagram based on the degree of enrichment is shown. The darker the color, the more genes are enriched in the pathway. Additionally, the PPI network identified in the gene list is depicted, with circles representing target proteins. The MCODE algorithm identifies subsets of interacting proteins, which are represented by nodes of the same color. The right side of the diagram displays the MCODE component list identified in the gene list. Terms belonging to the same cluster are closely related to each other. The degree of enrichment (P-value) indicates that a higher number of included genes corresponds to a more significant P-value. For clarity, only one term label per cluster is shown. The size of nodes in the network diagram reflects their degree, with larger nodes indicating higher degree values and shorter distances between functionally similar channels.

**Figure 11:**
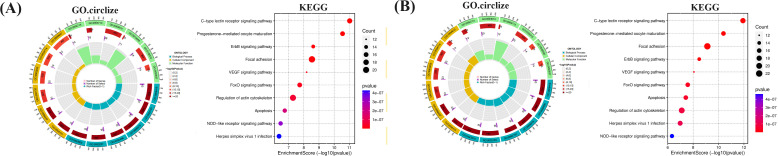
The functional process and molecular pathway analysis. **(A)** MXSG-mix1, **(B)** MXSG-mix2. The top 10 GoO functions are ranked according to their P value. The bar chart shows the bars representing the GO functions that enrich cross-target proteins. The y-axis is sorted according to P value. The abscissa represents P-value, while the x-axis indicates the number of cross-targets present in each GO function. Additionally, the KEGG: pathways are shown based on their P-value, with color shading indicating the significance of the P-value and the size representing the number of cross-targets in each pathway. The figure also highlights the top 3 GO terms associated with the 10 Hubba genes and their corresponding KEGG pathway.

To further investigate the relationship between enriched terms, the Kappa score was computed as a metric for assessing word similarity. Subsequently, an enriched term similarity network was constructed (Additional file 7-8). For visualization purposes, Cytoscape 3.7.1 was employed. Within this network, each node represents a term (**Figure 10**) and is evaluated based on its cluster ID and p-value (importance). Nodes are connected if the term similarity exceeds a threshold of κ > 0.3, thereby forming a network of enriched terms (Additional file 9). It is evident that terms belonging to the same cluster exhibit closer associations with one another. The size of nodes in the network reflects their degree of enrichment (*p*-value). Notably, larger nodes indicate higher degree values and shorter distances between functionally similar channels. In order to enhance clarity, only one term label is displayed for each cluster. Based on the analysis findings, it has been determined that the target genes involved in host defense and stress responses play important roles in the pathogenicity of IBV. Gene ontology (GO) terms suggest that these genes participate in biological processes and molecular functions related to host defense and stress responses (**Tables 5**, **6**). The Kyoto Encyclopedia of Genes and Genomes (KEGG) results demonstrate significant enrichment of target genes across various pathways, with higher values indicating a greater level of enrichment. The size of the dots in the KEGG plot represents the number of target genes present in each pathway. This study reveals distinct levels of enrichment for both drugs within various signaling pathways, including but not limited to C-type lectin receptor signaling pathway, Progesterone-mediated oocyte maturation, ErbB signaling pathway, Focal adhesion, VEGF signaling pathway, FoxO signaling pathway, Regulation of actin cytoskeleton, Apoptosis, NOD-like receptor signaling pathway, and Herpes simplex virus 1 infection pathway. The degree of enrichment varies depending on the P-value results (**Figure 11**).

**Table 5:** Analysis of the MXSG-mix1 GO circle chart.

Ontology	ID	Description
BP	GO:0009410	Response to xenobiotic stimulus
BP	GO:0062197	Cellular response to chemical stress
BP	GO:0006979	Response to oxidative stress
BP	GO:0032496	Response to lipopolysaccharide
BP	GO:0002237	Response to molecule of bacterial origin
BP	GO:0050900	Leukocyte migration
CC	GO:0045121	Membrane raft
CC	GO:0098857	Membrane microdomain
CC	GO:0031983	Vesicle lumen
CC	GO:0060205	Cytoplasmic vesicle lumen
CC	GO:0034774	Secretory granule lumen
CC	GO:0009897	External side of plasma membrane
MF	GO:0004715	Non-membrane spanning protein tyrosine kinase activity
MF	GO:0004713	Protein tyrosine kinase activity
MF	GO:0004674	Protein serine/threonine kinase activity
MF	GO:0019956	Chemokine binding
MF	GO:0106310	Protein serine kinase activity
MF	GO:0020037	Heme binding

**Table 6:** Analysis of the MXSG-mix2 GOcircle chart.

Ontology	ID	Description
BP	GO:0009410	Response to xenobiotic stimulus
BP	GO:0050900	Leukocyte migration
BP	GO:0006979	Response to oxidative stress
BP	GO:0062197	Cellular response to chemical stress
BP	GO:0032103	Positive regulation of response to external stimulus
BP	GO:0043410	Positive regulation of MAPK cascade
CC	GO:0045121	Membrane raft
CC	GO:0098857	Membrane microdomain
CC	GO:0031983	Vesicle lumen
CC	GO:0060205	Cytoplasmic vesicle lumen
CC	GO:0034774	Secretory granule lumen
CC	GO:1904813	Ficolin-1-rich granule lumen
MF	GO:0004713	Protein tyrosine kinase activity
MF	GO:0004674	Protein serine/threonine kinase activity
MF	GO:0004715	Non-membrane spanning protein tyrosine kinase activity
MF	GO:0106310	Protein serine kinase activity
MF	GO:0002020	Protease binding
MF	GO:0020037	Heme binding

**Figure 12:**
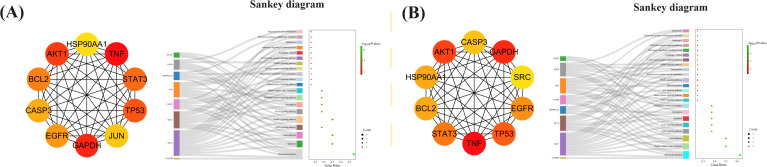
The Sankey diagram illustrates the correspondence between the top 10 genes based on MNC scores (left) and the enriched GO functions and KEGG pathways (right). **(A, B)** present two sets of such Sankey diagrams and associated bubble charts. In each sub - figure, the bubble chart displays functional descriptions, with color depth indicating the P - value and diameter size representing the number of cross - targets within the current GO function or KEGG pathway.

**Figure 13:**
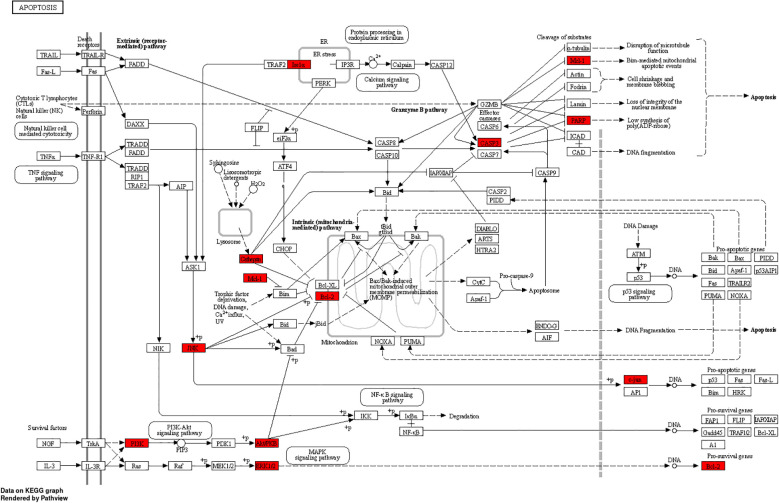
MXSG-mix is involved in the cell apoptosis pathway, and the proteins regulated by MXSG-mix are highlighted in red.

To detect densely connected regions in large PPI networks that potentially represent molecular complexes, a method based on vertex weighting of local neighborhood density and outward traversal of local dense seed proteins was employed. This method effectively isolates dense regions according to specified parameters and surpasses other graph clustering methods in performance. It allows for precise adjustment of clusters of interest without considering the rest of the network, enabling examination of cluster interconnectivity associated with protein networks. In order to provide a more intuitive representation of protein interactions, the topological features of nodes in the PPI network were analyzed (Peng et al., 2022). By utilizing the MNC, 10 key targets that could be used for MXSG-mix treatment against IBV were screened and hypothesized to play a crucial role (Additional file 10). The Sankey diagram depicted in **Figure 12** illustrates that the key targets identified for MXSG-mix1 are mainly involved in Salmonella infection, apoptosis, and focal adhesion signaling pathways. On the contrary, MXSG-mix2 primarily targets Salmonella infection, herpes simplex virus I infection, and focal adhesion signaling pathways. AKT1, BCL2, and CASP3 were identified as common targets with significant involvement in these pathways. The MNC algorithm was employed to identify the top three key target proteins of both drugs by integrating KEGG results and conducting a comprehensive analysis of the Sankey diagram. Subsequently, the impact of drugs on these key proteins was validated through the apoptosis pathway. As a future research direction, emphasis will be placed on investigating the role of the apoptosis pathway (**Figure 13**).

### 3.5 Validation of primary targets

**Figure 14:**
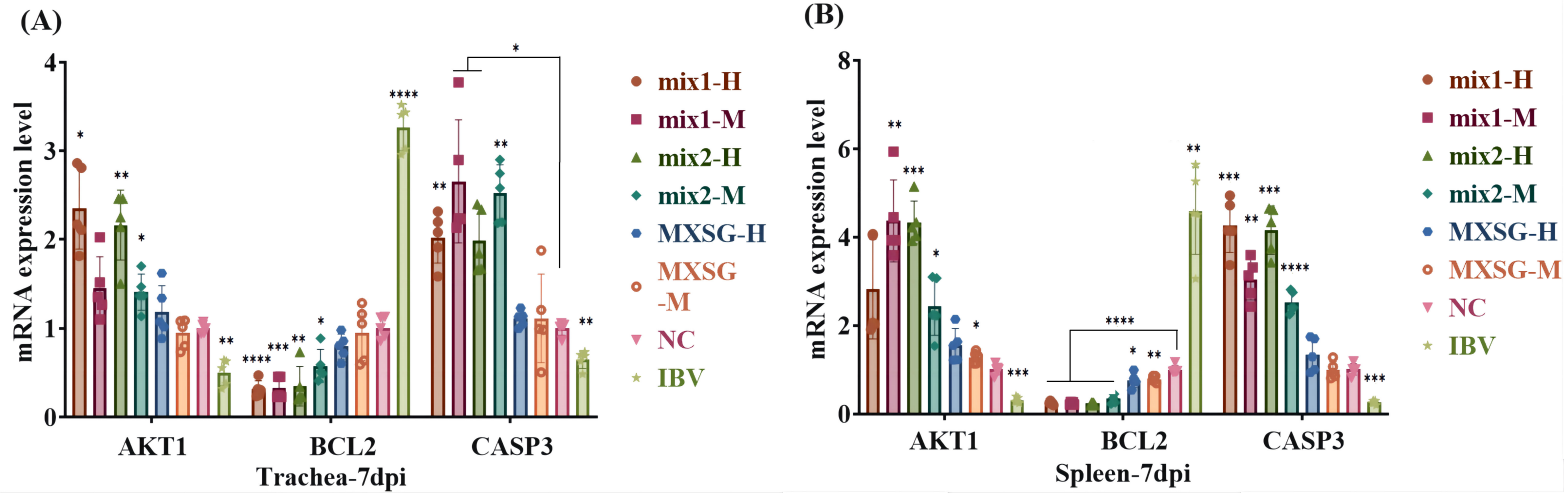
Expression levels of Hubba gene mRNA: **(A)** Tratea mRNA expression level; **(B)** Spleen mRNA expression level.

The validation of primary targets was conducted through qPCR amplification of chicken trachea and spleen tissue samples. Standard curve, amplification curve, and melting curve were obtained for each gene. The amplification efficiency for each gene was deemed satisfactory, exhibiting specific peaks with no instances of primer dimer or non-specific amplification. In **Figure 14**, it is evident that in the IBV group, the expression levels of AKT1 and CASP3 in the trachea were significantly reduced, while the expression level of BCL2 was increased compared to the NC group. In the MXSG group, there were no significant differences observed compared to the NC group, except for an increase in BCL2 index in the spleen. These findings suggest that AKT1, BCL2, and CASP3 are key targets influenced by alterations in MXSG levels. Furthermore, in the group receiving MXSG-mix treatment, there was an upregulation of AKT1 and CASP3 expression in the trachea along with a downregulation of BCL2 expression compared to the NC group.

**Figure 15:**
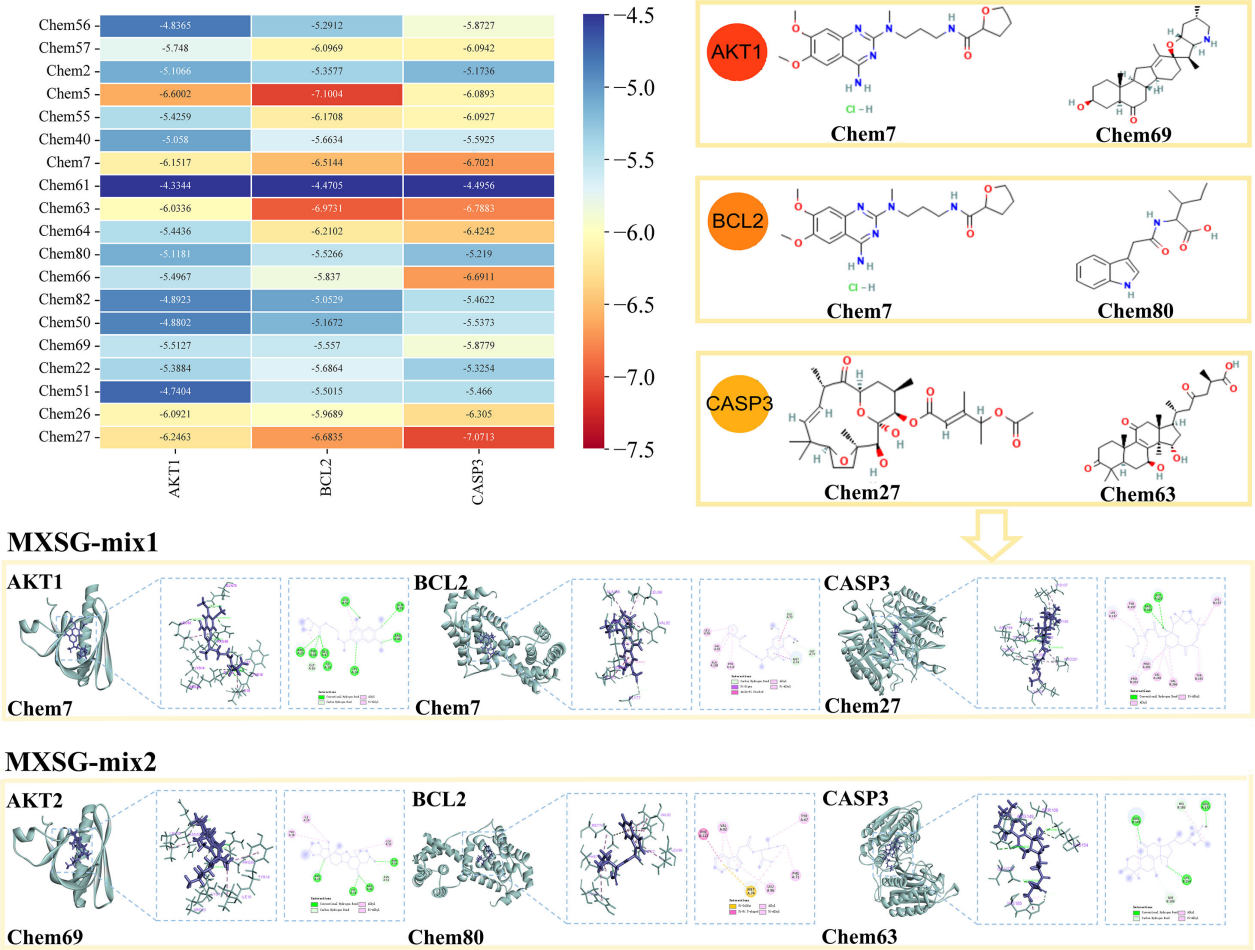
Heat maps of binding energy scores and molecular docking presentation of key compounds involved in major target proteins in the MXSG-mix group.

The study employed network pharmacology and organized the findings into **Table 7**, in order to identify compounds that induce alterations in key targets. Subsequently, batch molecular docking on AKT1, BCL2, and CASP3 was conducted using the Discovery software. The molecular docking scores were then visualized using R language’s Heatmap package, revealing that the MXSG-mix1 group was primarily influenced by Chem7 (Alfuzosin hydrochloride) and Chem27 (Tonantzitlolone B), while the MXSG-mix2 group was mainly affected by Chem69 (Peimisine), Chem80 (Indole-3-acetyl-L-isoleucine), and Chem63 (Ganoderic Acid A). For further analysis, Pymol software was used to visually examine the binding modes and sites of the main compounds with the key targets based on their highest absolute scores corresponding to different prescriptions in **Table 7** (**Figure 15**).

**Table 7:** Key compounds involved in the major target proteins in the MXSG-mix group.

Group	Mol	Name
mix-1	Chem2	2-(1-hydroxy-1-methoxy-3-methylbutyl)-6-methyl-5H-[1,3]dioxolo[4,5-c]pyridin-4-one
mix-1	Chem7	Alfuzosin hydrochloride
mix-1	Chem22	Platyphylline
mix-1	Chem26	Tamoxifen
mix-1	Chem40	Adenosine 3’,5’-cyclicmonophosphate
mix-1	Chem51	Rofecoxib
mix-1	Chem7	Alfuzosin hydrochloride
mix-1	Chem50	Parietinic acid
mix-1	Chem5	3,4-Di-O-caffeoylquinic acid
mix-1	Chem27	Tonantzitlolone B
mix-2	Chem55	(7E)-3-Isobutyl-4,5,8,12,12-pentamethyl-3,3a,4,6a,9,10,10a,13a,14,15-decahydro-1H-[1,3]dioxolo[7,8]cycloundeca[1,2-d]isoindole-1,16(2H)-dione
mix-2	Chem2	2-(1-hydroxy-1-methoxy-3-methylbutyl)-6-methyl-5H-[1,3]dioxolo[4,5-c]pyridin-4-one
mix-2	Chem64	Imperialine
mix-2	Chem66	Korseveriline
mix-2	Chem69	Peimisine
mix-2	Chem76	Adenosine 3’:5’-cyclicmonophosphate
mix-2	Chem80	Indole-3-acetyl-L-isoleucine; PlaSMA ID-525
mix-2	Chem82	Naringenin
mix-2	Chem56	1-allyl-2-hydroxy-7,8,9,10-tetrahydroazepino[2,1-b]quinazolin-12(6H)-one
mix-2	Chem57	1-Cyclohexene-1-carboxylic acid, 6-[3-(beta-D-glucopyranosyloxy)butyl]-5,5-dimethyl-3-oxo-
mix-2	Chem5	3,4-Di-O-caffeoylquinic acid
mix-2	Chem61	Caffeine-d3
mix-2	Chem63	Ganoderic Acid A

## 4 Discussion

IBV, a gamma coronavirus, undergoes frequent mutation, resulting in the emergence of new strains. This constant mutation poses significant challenges for prevention and control as cross-protection between vaccines and field strains is generally ineffective. Therefore, the development of potent antiviral therapies is crucial. Previous studies have demonstrated that plant extracts have substantial antiviral effects against IBV (Zhang et al., 2018; Jackwood et al., 2010; Yin et al., 2011; Lelesius et al., 2019; Chen et al., 2014). These extracts can inhibit viral replication, disrupt the virus’s structure, influence virus adsorption, and modulate host immune responses. In terms of inhibiting viral replication, plant extracts can positively impact the host’s respiratory system by promoting mucolysis and bronchiectasis (Wani et al., 2021). Specific extracts, such as terpenoids 1, 8-eucalyptol, (-) -α-pinene, and (-) -β-pinene, target the essential IBV-N protein involved in virus replication (Yang et al., 2010, 2011). Disrupting the structure of the virus represents an additional antiviral mechanism. For example, black elderberry extract has the ability to render the virus non-infectious by inducing disintegration of its structure (Chen et al., 2014). In terms of its impact on virus adsorption, extracts from Allium sativum, Houttuynia cordata, and Sambucus nigra may exert a direct virucidal effect by inactivating viral envelope structures that are necessary for the virus’s adsorption or entry into host cells (Yin et al., 2011). Lastly, some extracts can also modulate host immune responses. Isatidis root polysaccharide and andrographolide, for instance, possess the capability to suppress viral activity through regulation of inflammation and programmed cell death pathways (Xiang et al., 2023; Shen et al., 2023). However, it is important to note that most existing research primarily focuses on validating the efficacy of these therapies *in vitro* or *in vivo* settings. A comprehensive evaluation regarding their overall therapeutic impact on the human body remains largely unexplored.

Traditional Chinese veterinary medicine relies on clinical experience and pharmacological effects to design drugs. Diagnosis and syndrome differentiation are used for treatment planning and selecting the appropriate traditional Chinese medicine formulas. However, the use of big data from the TCM Heritage Computing platform (V3.0) now allows researchers to efficiently screen drugs or prescriptions with specific pharmacological activities, facilitating the development of new veterinary drugs through integrating existing information on TCM prescriptions and veterinary medications. This not only supports the research and development of new drugs but also reduces reliance on experimental animals while enhancing animal welfare. MXSG is a traditional Chinese medicine formula primarily employed for heat clearance, phlegm elimination, and cough relief. Recent studies have indicated that MXSG demonstrates therapeutic effectiveness against infectious bronchitis (IB) in chickens. MXSG, a compound derived from traditional Chinese medicine, has the potential to inhibit virus replication (Cheng et al., 2023; Hsieh et al., 2012) and improve the pathological damage in diseased lung tissue (Wang et al., 2020), effectively treating symptoms similar to those caused by IBV (Li et al., 2021; Gajewski et al., 2021; Zeng et al., 2021). Additionally, MXSG has been identified as a potential drug for combating coronavirus during the COVID-19 pandemic. However, further scientific research is necessary to elucidate its specific pharmacological mechanism, optimal dosage, and application strategy. Some modified formulations based on MXSG have shown enhanced therapeutic effects in studies targeting symptoms resembling IBV (Zhu et al., 2023; Dai et al., 2013). In this study, commercial TCM prescription formulations for IBV-like symptoms were compiled using various TCM prescription databases. A dataset of TCM prescriptions for IBV disease was created by leveraging the anti-IBV potential of MXSG. The TCMICS V3.0 platform was utilized to generate two types mixtures called MXSG-mix1 and MXSG-mix2 with additions and reductions (**Figure 1**). This marks the first application of the platform within veterinary medicine for drug improvement.

In order to assess the safety and efficacy of the drugs, the therapeutic effects were initially compared in chicken embryos. It was discovered that MXSG-mix1-H was the highest effectiveness in inhibiting IBV-M41 on embryos (EI: 3175.458 ± 555.279), while also exhibiting minimal toxicity towards chick embryos. Furthermore, it significantly reduced chick embryo mortality and improved the condition of chick embryo dysplasia, thereby highlighting the antiviral potential of MXSG. In this study, the MXSG-mix2-H group demonstrated even more pronounced anti-IBV properties, resulting in reduced morbidity, suppressed IBV RNA expression levels, improved cure rate of IBV-infected chickens, and promoted body growth (**Figure 3B**). Specifically, all MXSG-mix2-H groups exhibited lower clinical scores compared to the IBV group, indicating strong antiviral effects (**Figure 3A**). Additionally, the MXSG-mix2-H group showed significantly reduced inhibition of IBV RNA expression when compared to the IBV group (**Figure 6**). Tracheal protection was higher in the MXSG-mix2-H group than in the MXSG-positive group as evidenced by tissue lesion recovery (**Figure 5**; **Table 3**). Virus infection often leads to changes in various physiological indicators. By comparing blood routine indicators, inflammatory markers, and antioxidant markers before and after drug treatment, the efficacy of the drugs was evaluated. It was observed that different traditional Chinese medicine compounds had varying effects on the blood indicators of chickens at different collection times, with most of the traditional Chinese medicine treatment groups showing effectiveness at 3 dpi. The effects on individual measures persisted for up to 1 week after discontinuation of drug administration (**Table 4**). Based on the six anti-inflammatory indexes, the MXSG-mix1-M group exhibited superior anti-inflammatory effects compared to the other two TCM formulas (**Figure 7**). The MXSG-mix group outperformed the positive control drugs in terms of five antioxidant indexes (**Figure 8**). The level of immune organ index can serve as an indicator of the body’s immune strength to some extent, making it a commonly used measure for evaluating immune status. The spleen, an important peripheral immune organ, plays a crucial role in eliminating foreign substances by promoting lymphocyte proliferation and cytokine synthesis (Shojadoost et al., 2019; Zhang et al., 2016). The study revealed that the MXSG-mix group exhibited a significantly enhanced ability to stimulate immune organs, particularly the spleen. This suggests that the MXSG-mix group has the potential to augment the immune response of chickens and potentially mitigate viral activity, indicating a stronger immune response compared to MXSG (**Figure 4**). Antibody levels serve as crucial indicators of humoral immunity during infection. Humoral responses are considered vital in preventing IBV (Chhabra et al., 2015). It was observed that the MXSG-mix2-H group had higher antibody levels than the IBV group at 7 dpi, while the MXSG-mix1-H group exhibited the highest antibody levels at 14 dpi. Both findings indicate an improved humoral response in chickens compared to MXSG (**Figure 9**). Considering these indicators, it is evident that developing MXSG with added and reduced mixtures, particularly focusing on the MXSG-mix2-H group, yields superior antiviral effects. Furthermore, it influences both the chicken’s immune system and growth performance, leading to an overall improvement in their health status, including nutritional and disease aspects.

The application of LC-MS is essential for identifying additives and various pharmaceutical components in MXSG, showcasing its remarkable precision, sensitivity, and throughput. This cutting-edge technology plays a pivotal role in unraveling the composition of traditional Chinese medicine compounds. By harnessing data obtained from LC-MS analysis, network pharmacology can predict and elucidate the comprehensive efficacy as well as underlying mechanisms of these compounds. This research strategy combines state-of-the-art instrumental analysis techniques with systems biology methods to provide novel insights and tools for advancing research on traditional Chinese medicine compounds. To illustrate the advantages of MXSG addition and subtraction mixture compared with MSXG, liquid chromatography tandem mass spectrometry was used to identify MXSG along with its addition and subtraction mixture (**Figure 10**). The integrated composition data were analyzed to screen out the added drug components and targets within the traditional Chinese medicine compound after addition and subtraction. Drawing upon these component targets, a systems pharmacology approach was employed to predict and clarify the potential molecular mechanisms of the active substances within the MXSG-mix on IBV. Through compound target pathway and target pathway network analysis, the key genes within the effective active ingredient-target network of MXSG-mix were discerned, which aided in constructing a protein interaction network (**Figure 11**).

In the results of GO enrichment analysis, both MXSG-mix1 and MXSG-mix2 exhibited similar GO outcomes. These results impacted molecules involved in various processes, including xenostimuli response, leukocyte migration, and oxidative stress response. Additionally, they regulated the binding of cellular components such as membrane rafts, membrane microdomains, and vesicle compartments. The function of these products is to further regulate biological processes, specifically related to non-membrane transmembrane protein tyrosine kinase activity, protein tyrosine kinase activity, protein serine/threonine kinase activity, etc (**Figures 12**, **13**). This regulation aims to control IBV and achieve the desired effect under investigation. In the animal studies at 3 dpi, the MXSG-mix1 group exhibited a significant increase in NO or T-AOC content. Similarly, the MXSG-mix2-H group was able to prevent a decrease in XOD and MDA activities at 3 dpi before other groups. By 7 dpi, notable differences was observed between the MXSG-mix group and the IBV group. By 14 dpi, MDA levels along with T-AOC, and SOD indices were comparable to those of the control group indicating recovery from oxidative stress response as indicated by GO results. White blood cells (WBC) are categorized into granulocytes, lymphocytes, and monocytes, playing a crucial role in defending against foreign invaders such as pathogenic microorganisms. Fluctuations in the peripheral blood WBC count can affect their functionality as they are involved in pathogen elimination, allergen removal, immune responses, and antibody production through processes like exudation, chemotaxis, and phagocytosis. Inflammation often leads to changes in the peripheral blood WBC count. The GO results suggest that MXSG-mix may induce WBC migration, which is supported by significant differences between the MXSG-mix group and the IBV group during animal testing of blood counts, inflammatory factors, and antibody production levels.

Studies have shown that viruses induce apoptosis to facilitate efficient viral dissemination while evading excessive inflammatory responses and immune system activation (Zhou et al., 2017). Viral infection often causes cell damage, triggering apoptotic signaling pathways. BCL2, a pivotal gene inhibiting apoptosis, plays a critical role in maintaining the equilibrium of BCL2 family proteins. When viral infection causes cell damage, this equilibrium is disrupted, resulting in an increase in pro-apoptotic proteins and subsequent cellular apoptosis. Moreover, viral infection may activate caspase enzymes, with CASP3 being involved in the regulation of the AKT pathway. Experimental evidence suggests a correlation between CASP3 expression levels and AKT phosphorylation. Increased expression of CASP3 may further activate the AKT pathway, thereby promoting enhanced activation of the apoptotic pathway. IBV infection induces apoptosis in CEK cells and significantly reduces the expression of Bcl-2 protein, while also increasing Caspase 3 mRNA expression levels (Fung et al., 2014; Chhabra et al., 2016; Chen et al., 2019a; Liao et al., 2013). Some antiviral drugs can regulate cell apoptosis to inhibit viral proliferation. In this study, KEGG enrichment analysis revealed several signaling pathways (e.g., Apoptosis, Salmonella in fections, Focal adhesion) that are strongly associated with the development and metastasis of infectious bronchitis in chickens (**Figures 13**, **14**). To provide a more intuitive representation of protein interactions, the topological features of nodes in the PPI network were analyzed. As a result, based on MNC and playing a pivotal role, 10 key targets were identified and assumed for MXSG-mix for IBV. Among these targets, AKT1, BCL2, and CASP3 emerged as the top three common core genes. By combining KEGG results with comprehensive analysis using the Sankey diagram, the first three key target proteins of two drugs were successfully identified through implementation of the MNC algorithm. The impact of drugs on these crucial proteins was confirmed by evaluating their effects on the apoptosis pathway. The expression levels of AKT1, BCL2, and CASP3 genes were compared between the trachea (the primary target organ for infectious bronchitis virus) and the spleen (the immune organ most susceptible to drug administration). Statistical analysis was conducted to investigate the relationship between the control group (NC) and drug group (**Figure 15**). Apart from the MXSG-M group of the spleen, none of the other MXSG treatment results showed significant differences compared to the control group. This study provides compelling evidence that MXSG-mix, a specific treatment for IBV, may exert its function by modulating the expression of three key genes: AKT1, BCL2, and CASP3. These genes play crucial roles in cellular maintenance and survival. AKT1 is a protein kinase that regulates cell growth and proliferation, while BCL2 is a protein that prevents cell death or apoptosis (Guo et al., 2018). CASP3, on the other hand, is a protease responsible for executing apoptosis. It was discovered that MXSG-mix might promote Bcl-2 mRNA expression and reduce Caspase 3 mRNA expression by attenuating the AKT signal pathway, thereby inhibiting cell apoptosis and weakening IBV infection to achieve an improved antiviral effect. However, further experimentation is required to validate this proposed molecular mechanism. This could involve manipulating gene expression or using specific inhibitors to determine if they are indeed the primary targets of MXSG-mix in its anti-IBV action. Overall, this study identifies potential targets for anti-IBV treatments and offers a promising avenue for future research into the molecular mechanisms of MXSG-mix.

## 5 Conclusion

In this study, the deep mining technology of TCMICS was utilized to construct a clinical prescription dataset for treating IBV in order to generate an augmenting and reducing mixture of Maxing Shigan Decoction (MXSG-mix). The effectiveness of MXSG-mix was demonstrated through both an egg experiment and an *in vivo* experiment. Subsequently, the change in composition of MSXG mixture was determined using LC-MS, and the molecular mechanism underlying MXSG-mix’s action against IBV was preliminarily explored using network pharmacology. These findings were further validated through animal experiments. The integration of TCMICS, LC-MS, and network pharmacology in data analysis provides a novel perspective for advancing Chinese veterinary medicine. MXSG-mix shows promising potential as a highly effective treatment for chicken infectious bronchitis that may surpass traditional MXSG.

## Data Availability

The datasets presented in this study can be found in online repositories. The names of the repository/repositories and accession number(s) can be found in the article/**Supplementary Material**.

## Glossary

BPI: Base Peak Intensity
CBC: complete blood count
DDA: data-dependent acquisition
dpi: day post-infection
EID50: 50 percent Egg Infectious Dose
ESI: electrospray ionization
EI: Embryo Index
GRAN%: granulocyte percentage
HGB: hemoglobin content
HCT: hematocrit
IBV: Infectious bronchitis virus
LYM%: lymphocyte percentage
MCH: mean corpuscular hemoglobin
MCHC: mean corpuscular hemoglobin concentration
MCV: mean corpuscular volume
MID%: percentage of intermediate cells
MNC: maximum neighborhood component
MPV: mean platelet volume
MXSG: Maxing Shigan Decoction
PCT: plateletcrit
PDW: platelet distribution width
PPI: protein-protein interaction
RBC: red blood cell count
TCM: Traditional Chinese Medicine
TCMICS: Traditional Chinese Medicine Inheritance Computing Platform
WBC: white blood cell count
